# Accessing equitable menopause care in the contemporary NHS: a qualitative study of women's experiences

**DOI:** 10.3399/BJGP.2024.0781

**Published:** 2025-11-04

**Authors:** Abi Eccles, Sabrina Keating, Claire Mann, Lisa Shah, Jeremy Dale, Patricia Apenteng, Neelam Heera, Nina Kuypers, Lynn Tatnell, Sarah Hillman

**Affiliations:** 1 Nuffield Department of Primary Care Health Sciences, University of Oxford, Oxford, UK; 2 University of Warwick Medical School, University of Warwick, Coventry, UK; 3 Nuffield Department of Primary Care Health Sciences, University of Oxford, Oxford, UK; 4 Department of Applied Health Sciences, University of Birmingham, Birmingham, UK

**Keywords:** menopause, inequalities, hormone replacement therapy, qualitative research, primary health care, socioeconomic status

## Abstract

**Background:**

Women from lower socioeconomic status and minority ethnic backgrounds have earlier onset and more complex menopause symptoms. Hormone replacement therapy (HRT) has grown in popularity in recent years, however there are stark disparities in those who access HRT. Rates of use in deprived areas and for Black and Asian women are significantly lower than that of White women and those in more affluent areas.

**Aim:**

To explore women’s experiences of menopause and accessing primary care, as well as how perceptions and approaches may be shaped by cultural norms, to gain deeper understanding of factors shaping approaches to managing menopause and HRT prescribing patterns.

**Design & setting:**

Qualitative study with women recruited from general practice and community networks.

**Method:**

In-depth semi-structured interviews and focus groups with women experiencing menopause (*n* = 40) were conducted between October 2023 and March 2024. Purposive sampling allowed a breadth of experiences and thematic analysis was conducted.

**Results:**

Three themes were developed in relation to women’s experiences of accessing menopause care: 1) ‘Contemporary contexts’ shaped women’s experiences; managing menopause alongside high workloads and caring responsibilities posed challenges perceived as distinct from previous generations; and there was heightened awareness, reduced stigma, and mixed views regarding HRT; 2) ‘How menopause care is experienced’ demonstrated how consultations about menopause were emotionally charged, many felt they would have to advocate for HRT (if they wanted it), and some felt frustrated with the lack of options available; and 3) ‘Cultural and economic background influences on menopause help seeking’ included how some women from Black or Asian backgrounds did not discuss menopause within their communities. Mistrust of medical institutions and treatments, as well as lack of representation, was problematic for Black and Asian participants. Some women worried about stereotyping during consultations.

**Conclusion:**

The findings outline how menopause may be particularly disruptive to modern women, demonstrate how women are often dissatisfied with the options available, and highlight key areas, such as communication about HRT benefits and/or risks, which could be improved in primary care settings.

## How this fits in

Menopause awareness has increased in recent years, as well as hormone replacement therapy (HRT) use, however this has not been experienced equally. Cultural influences such as stigma, preferences for non-medical approaches, perceptions of ailments appropriate for health care, and lack of representation work against women seeking help. This study found GPs should not assume all women who would benefit from HRT will advocate for it. GPs should initiate discussions about potential HRT, as well as other approaches, with all presenting women who may benefit.

## Introduction

Menopause is a biological stage when menstruation ceases and oestrogen levels decrease. Approximately 85% of women report symptoms of varying severity including hot flushes and joint pain.^1,2^ The UK Government’s *Women’s Health Strategy* reported that only 9% of women felt they had sufficient menopause information and only 64% felt comfortable talking to healthcare professionals about menopause.^3^ Menopause in women from lower socioeconomic status and minority ethnic backgrounds could have a particularly detrimental impact, as menopause onset is likely to be earlier and symptoms are reported to be more complex.^4–6^ Additionally, South Asian and Black individuals and those from lower socioeconomic backgrounds have a higher risk of cardiovascular disease,^7,8^ further exacerbated by menopause,^9^ hence such women may stand to benefit most from menopause care.

Hormone replacement therapy (HRT) is often considered the first-line treatment,^10^ but there are a range of interventions that may alleviate menopause symptoms. National Institute for Health and Care Excellence guidelines refer to the importance of physical activity to maintain muscle mass and strength, but this is under the heading of ‘Information and support’ rather than ‘Discussing management options with people aged 40 or over’.^11^ ‘Hormone replacement therapy’, ‘Cognitive behavioural therapy’, and ‘Complementary therapies and unregulated preparations’ are included under management options (with guidelines stating that patients are informed that the safety and efficacy of approaches under the third option are unknown).

Popularity of HRT has grown in recent years with an estimated 2.3 million individuals prescribed HRT in England in 2022/2023, an increase of 70% from 2020/2021.^12^ However, there remain growing disparities in who accesses HRT; in 2020, prescriptions were 29% lower in practices in the most deprived parts of England compared to those in more affluent areas (per registered female patients aged ≥40 years),^13^ a difference that widened further during 2022/2023.^12^ Disparities also exist when considering the ethnicities of women (aged between 45 and 55 years), with the rates of use in Black (5.2%) and Asian women (6.2%) being significantly lower than White women (23.3%).^14^

The reasons for such inequalities in menopause care are poorly understood. Qualitative investigation allows in-depth exploration of women’s perceptions and experiences, which in turn may provide insights behind some of the disparities. In this study, women’s experiences of menopause and accessing primary care were explored, as well as how perceptions and approaches may be shaped by cultural norms. This study aimed to gain deeper understanding of factors shaping approaches to managing menopause and HRT prescribing patterns through undertaking a qualitative investigation.

## Method

### Study design

This is an NIHR funded study (Research for Patient Benefit) MenopauseGAP, which examines menopause care in contemporary England. It reports findings from the women’s data in line with the Standards for Reporting Qualitative Research guidelines.^15^ Findings from the healthcare professional data are reported elsewhere ^16^ and complement this study, providing further insights from clinicians’ perspectives. To investigate women’s experiences, we combined a series of in-depth semi-structured interviews and focus groups conducted between October 2023 and March 2024. Topic guides were designed to explore women’s experiences of menopause, accessing primary care, and their approaches to managing menopause, as well as their views on how social backgrounds shape experiences (see Supplementary Box S1).

We worked closely with women who are community leaders and/or had experienced menopause symptoms. They facilitated recruitment via their established networks, commented on findings, helped develop recruitment materials, and advised on terminology. They also advised us, to ensure inclusivity, we ought to conduct both focus groups and interviews, thus allowing participants to take part in their preferred way. Four of the researchers conducted data collection via telephone or video-call.

### Study participants

Eligible women lived in England, were aged between 40 and 60 years, symptomatic within the previous 3 years, and able to provide consent. Individuals who had had a hysterectomy or identified as transgender were excluded due to likelihood of having alternative experiences and access to care.

To explore mediating social factors shaping women’s experience a purposive sampling strategy was adopted based on the known disparities in HRT use. This enabled recruitment of a diverse sample in terms of ethnicity, deprivation, and HRT use (see Table 1). Participants received a shopping voucher and were recruited via West Midlands general practices and community leaders’ networks. We conducted four focus groups and 27 interviews. Participants within each focus group varied in terms of HRT use: one focus group was with Black women; one was with South Asian women; and two were with women from a range of ethnicities.

**Table 1.** Participant characteristics

Characteristic	*n* (%)
**Total**	40
**Data collection method**	
Focus group	13 (33)
Interview	27 (68)
**Recruitment setting**	
General practice	15 (38)
Community	25 (63)
**English indices of deprivation, decile**	
1 (most deprived)	5 (13)
2	5 (13)
3	3 (8)
4	6 (15)
5	1 (3)
6	3 (8)
7	1 (3)
8	8 (20)
9	1 (3)
10 (least deprived)	4 (10)
unclear	3 (8)
**Ethnicity**	
South Asian	13 (33)
Black	7 (18)
White British/Irish/Other	18 (45)
Mixed	2 (5)
**HRT use**	
Never used	17 (43)
Currently using	14 (35)
Previously used	8 (20)
Use not recorded	1 (3)

HRT = hormone replacement therapy.

### Data analysis

Interviews and focus groups were audio-recorded and transcribed verbatim, anonymised, and uploaded to NVivo (version 11). Fieldwork researchers were all women from a range of ethnicities and career stages; four were experienced in women’s health qualitative research and two were practising GPs. They independently carried out familiarisation and initial coding, guided by reflexive thematic analysis.^17^ A coding framework was developed iteratively and scrutinised over a series of meetings. Interview data organised into codes relevant to key themes were exported and analysed using the one sheet of paper method.^18^ Two researchers ( mapped and grouped content, identified patterns, and created a conceptual map for each key theme. These findings were further scrutinised and developed with input from the study’s public representatives. Quotes are presented to illustrate findings, with extended quotes available in Supplementary Box S2.

## Results

**Figure 1.**
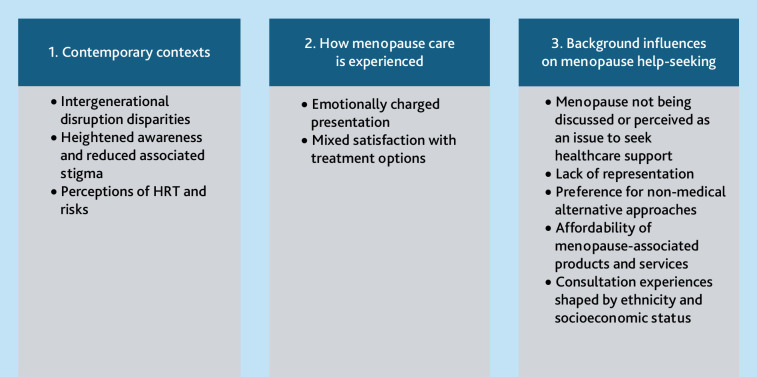
Themes grouped into overarching areas. HRT = hormone replacement therapy.

Participants described a range of menopause-related symptoms, including brain fog, irritability, fatigue, bloating, joint pains, hot flushes, vaginal dryness, and loss of libido. The impact on everyday life could be severe, with participants attributing long-term sick leave, relationship breakdowns, and feeling suicidal to menopause. Women adopted various approaches to managing menopause and seeking help, and talked about how cultural norms can shape these. Three overarching thematic areas were found, each of which comprised several subthemes developed during analysis (Figure 1).

### Contemporary contexts

Three subthemes were developed deepening understandings of the contemporary contexts in which menopause care exists.

#### Intergenerational disruption disparities

Middle-aged women today appear to have heightened responsibilities compared with previous generations. Many women noted how their mother’s generation did not have so many competing priorities, such as heavy workloads and caring responsibilities (parents and children). Some suggested modern women have less time to manage the effects of menopause, as well as more responsibilities that symptoms disrupt:

*‘*[My mother] *was not managing a massive team with loads of responsibilities, and it was very, very different for her.’* (Interview [INT]15, White British woman)

*‘... it’s coinciding with a time when I’m very busy with a four-year-old, and also career wise* […] *with elderly parents, and juggling all of that ... ’* (Focus group [FG]05, Focus group of women of mixed ethnicities)

The findings demonstrate how menopause can disrupt modern women’s lives in ways that appear more notable than previous generations.

#### Heightened awareness and reduced associated stigma

Participants discussed increased menopause awareness within mainstream and social media in recent years, often referring to how ‘perimenopause’ was a term unknown to them until recently.

They were aware of a large variety of products claiming to help menopause symptoms. Some had tried many. Others felt cynical of the potential commercialisation of menopause or ‘meno-washing’, whereby prices of products are raised and unsubstantiated claims are made about their effects on menopause symptoms:

*‘Somebody’s just riding on the back of this, realising, you know, 51% of the population of women.’* (FG03, Focus group of women of mixed ethnicities)

*‘This bar of chocolate for £4 and it was for people who had menopause.’* (FG03, Focus group of women of mixed ethnicities)

Heightened awareness was often discussed in positive terms, but some felt overwhelmed and found assessing the credibility of information challenging. Many felt more reliable public health information was needed to prepare and support them:

*‘I’m trying to work out what’s more plausible and what’s more credible.’* (FG03, Focus group of women of mixed ethnicities)

*‘Nobody tells you, actually, them* [periods] *stopping is gonna be worse than them having, ever having started.’* (INT02, Asian Pakistani woman)

Several participants talked about menopause with peers to help them understand their symptoms and this sometimes influenced decisions about which strategies to adopt. There appeared to be greater openness to discuss menopause more freely, suggesting that stigma associated with menopause was reduced in contemporary contexts:

*‘Speaking to other women about this, about the anger, actually. And they said, “Oh, maybe it’s the rage.” And I said, “What’s the rage?” And they were, like, “The menopausal rage.”* […] *looking a bit deeper into it, and I thought, “Yeah. Yeah. Got all those things, I'll speak to, speak to a specialist about it.”’* (INT08, White British woman)

*‘I confided in a couple of friends, who, basically, just told me that HRT, for them, was like, one day they weren't coping, and they had all these symptoms, and then the next day, after HRT, it was like, light bulb moment. They just felt a lot better* […] *When I went to my doctors, I went in, and I decided, “I’m not, leaving without it.”’* (INT12, Black Caribbean woman)

Speaking with peers appeared to be a key catalyst for many women in trying a new approach or seeking help.

#### Perceptions of HRT and risks

Women’s attitudes towards the cancer risks of HRT varied, and were shaped by previous and current evidence, as well as their own personal circumstances. Some were confident they had a good understanding, whereas others felt fearful and uncertain, often referring to longstanding preconceptions that HRT had a high risk of cancer, borne from previous media discourses. Concerns remained, with some stating that links with cancer (however small) meant they would not consider HRT. Others were unsure about the validity of concerns and to what extent those risks still existed, leaving them feeling nervous about using HRT:

*‘... it* [HRT] *could increase the risk of cancer. I don't know whether that’s true, or whether it’s just myth* […] *it’s been a bit of a scary ... ’* (FG04, Asian woman)

Many talked about changes in discourse around cancer risks meant they had changed their minds about taking HRT:

*‘I’ve gone from, “Most definitely not. I’m not having HRT.”* […] *To having a complete 180* [...] *I went to my doctors, I went in, and I decided, “I’m not, I’m not leaving without it.”’* (INT12, Black Caribbean woman)

Some participants discussed how they thought a lot about the risks, weighed them up against the benefits, and made an informed decision to take HRT:

*‘They'll say, “Oh, I don't wanna take HRT, I might get breast cancer.” But then they're overweight, and they drink wine every night* […] *there’s risks everywhere ... ’* (INT14, White British woman)

*‘It was more important for me to manage the symptoms that I have at the moment, rather than worrying about the, worrying about the potential of getting cancer from it.* […] *the benefits of it outweighed the risk.’* (INT21, Black African woman)

### How menopause care is experienced

Women’s experiences of seeking help from their GPs about menopause varied, with some feeling satisfied and others experiencing frustration. Two related subthemes providing new insights into how menopause care is experienced were identified.

#### Emotionally charged presentation

Many participants sought help only when their symptoms were causing significant disruption to their lives. Some reported frustrations with delayed identification, misdiagnosis, and not being offered any advice or treatment:

*‘Everything got muddled, jumbled up into one big ball of experiences, and emotions. I just battled on myself, didn’t reach out then.’* (FG04, Asian woman)

Many participants talked about how they knew they wanted HRT before the consultation and felt they had to prepare to advocate for a prescription. They perceived GPs as potentially reluctant to prescribe due to various reasons including budgetary constraints, a woman’s age, and concerns about risks:

*‘If I went to the doctor and I asked for HRT, I think there’d be push back* […] *I don’t think I’ve got the energy yet to go and, sort of, make a case and be my own advocate.’* (FG04, Asian woman)

*‘I phoned up the GP. I was ready for a real argument about starting HRT ... ’* (INT14, White British woman)

Adding to the difficulty of such consultations, many talked about problems persistent with general practice more generally, such as difficulty getting an appointment, lack of continuity of care, and feeling like appointments were rushed. It was considered highly important that GPs listen to their concerns about menopause and provide long-term personalised holistic support.

#### Mixed satisfaction with treatment options

Many participants voiced frustrations with the treatment options available and how these were framed in consultations. Some thought their GP seemed reluctant to prescribe HRT, without any reasoning being communicated:

*‘It wasn't some recreational medication that I was asking for* […] *it felt very much, I was being told this is not good for me, but without* […] *the person listening to me ... ’* (INT02, Asian Pakistani woman)

Some preferred to manage their symptoms through complementary and alternative medicine (CAM) (including supplements) or lifestyle changes. Some described how their GP lacked familiarity with such approaches, thus they navigated menopause management without NHS services, relying on other sources like the internet, friends and family, and commercial providers:

*‘My doctor said, “I don't know. I can't tell you anything about those, I don't know anything about them.”’* (FG06, Black woman)

Sometimes women were offered contraception or antidepressants to manage menopause symptoms. Some were satisfied with these options, whereas others felt like they were not being taken seriously:

*‘They just fob you off with antidepressants. I mean, sometimes you just need a good chat and a good cry.’* (INT10, White British woman)

*‘Doctors just, like, pass you off with the pill instead of looking at alternative remedies or solutions.’* (INT24, White British woman)

These findings show how some women feel dissatisfied and face a set of challenges when accessing menopause care within general practice.

### Background influences on menopause help-seeking

Women discussed how cultural and economic backgrounds shape perceptions and experiences. During analysis we identified five subthemes (see Figure 1).

#### Menopause not being discussed or perceived as an issue to seek healthcare support

Although there appears to be heightened awareness and less stigma around menopause in recent years (see section above), many South Asian participants described how menopause was not openly discussed within their communities:

*‘... a lot of women probably wouldn’t talk about it, because it’s frowned upon* […] *keep your business to yourself* […] *don’t broadcast it.’* (INT05, Asian Indian woman)

*‘I had low fertility in my mid-30s, you know, I didn't tell any of my, you know, my community friends, my Asian friends* […] *because of, you know, what if it gets around the community* […] *There’s a big stigma attached to, you know, reproductive health, menopause health, mental health. And I think in some communities, I can speak for my own, sort of, ethnicity, Bangladeshi ethnicity, heritage, community.’* (FG05, Focus group of women of mixed ethnicities)

When women did discuss menopause, they noted how this was more likely to be within their own generation or with individuals external to their communities (for example, colleagues):

*‘I’ve heard of HRT, and I had a lot of White colleagues who used to say, “Oh, I’ve got, I’m on HRT* […] *but hardly ever had conversation with my Asian colleagues, friends, or family, relatives ... ’* (FG04, Asian woman)

Many Black and South Asian participants discussed how as menopause is perceived as a natural process, it is therefore often not considered a problem to be addressed:

*‘We come from Jamaican parents, our parental culture was simply, whatever came along, you just got on with it. But I don't even know if our mum even heard the word menopause ... ’* (FG06, Black woman)

*‘That perception in a certain community or communities that it’s not a illness, there’s, you know, you don't trouble the GP for it.’* (INT01, Asian Indian woman)

The findings demonstrate how stigma around menopause and perceptions that it is accepted as natural, rather than problematised as an ailment, can lead to fewer discussions and it not being considered something to seek health care for.

#### Lack of representation

Many participants talked about how the information they access about menopause is often unrelatable. They recognised that bodies differ due to ethnicities, and if they do not see women like themselves represented, they often disengaged:

*‘... so used to seeing these white pamphlets, you don't even read the writing, 'cause you're like, “Well, it’s not for me.” If I see a Black face on something, it instantly makes me curious* […] *there are differences in our bodies* […] *some races are more likely to get certain diseases, some races are more likely to be obese.’* (INT22, Woman of White and Black mixed ethnicity)

*‘You want an Asian understanding of it* […] *our bodies are, sort of, made different* […] *treatments might not be the same, you know, for me, compared to somebody who’s White British.’* (FG04, Asian woman)

For some participants, lack of representation was not only a problem within the information subject, but also in relation to the individual conveying information:

*‘If I’m wearing a hijab, and I’m on HRT, and I’m telling another woman wears a hijab too that HRT isn't a bad thing, she’s more likely to take it from that person.’* (INT02, Asian Pakistani woman)

Representation of relatable individuals appeared to be an important factor when engaging with materials and advice.

#### Preference for non-medical alternative approaches

It was common for South Asian and Black participants to describe how people within their communities would often opt for CAM approaches when managing physiological issues:

*‘West Indians generally are of the mindset that you can find a herbal thing* […] *the body that God has made for you was never designed to have all those drugs in so there has to be a more natural alternative* […] *drugs is not the first port of call.’* (FG06, Black woman)

*‘*[my mum] *would have her Indian stereotypes and assumptions about what’s okay to put into your body. In fact, she didn’t like me being on any kind of medication* […] *just wanna do things naturally ... ’* (FG04, Asian woman)

Some participants referred to how deeply ingrained mistrust of the medical institution is for some individuals within Black communities:

*‘That is quite deep ingrained in our, in our communities. That, that distrust and fear, that, you know, a lot of medicines may potentially not work for us, they’re not for us, they may harm us.’* (INT12, Black Caribbean woman)

*‘You get told, “Don't let them experiment on you,” because of what happened within the slave days. So there’s a real distrust of, “What are you going to be putting in me? I’m not being used as a …” or, “Will it cause me to, will my rates of cancer, the percentage be higher as a Black woman?”’* (INT16, Black African woman)

#### Affordability of menopause-associated products and services

Recent years have seen growing commercialisation of menopause products and services, and it was common for participants to highlight related expense. Some assessed costs as worthwhile, but others found them unaffordable. Other commitments, particularly for less affluent individuals, may take precedent over adopting self-care activities:

*‘People are just so busy trying to survive out there. They're just busy trying to get up, show up, make a couple of quid. Feed their family, go to bed* […] *to suggest to them that you, you can manage, you feel better by yoga, meditation, walking, self-care, blah, blah, blah. That would just seem ludicrous* […] *expensive.’* (INT12, Black Caribbean woman)

#### Consultation experiences shaped by ethnicity and socioeconomic status

Some women were concerned about the potential to be stereotyped by clinicians and some did not feel confident to advocate, which in turn could shape their conduct during consultations:

*‘... it’s always a battle to get what I need. You know, tall black woman* […] *It’s a stereotype really, that we'd manage ... ’* (INT21, Black African woman)

*‘They're working class or they're a different race or they haven't learned to articulate themselves, then they come out of there breathing a sigh of relief if they haven't irritated the doctor* […] *As a black woman, I will go in fully equipped* […] *I’m gonna be very calm* […] *you're not allowed to have the range of emotions necessarily, because you're then gonna get ostracised for being stereotypical of your race.’* (INT16, Black African woman)

Our findings demonstrate how concerns about stereotyping exist and some modify their behaviours within consultations because of this.

## Discussion

### Summary

This study provides novel insights into how women experience menopause care and the ways in which cultural norms may shape their perceptions and approaches. These insights focused around three overarching themes, including contemporary contexts; how menopause care is experienced; and cultural and economic background influences on menopause help-seeking.

The findings reveal some of the context behind current heightened awareness of menopause and how women’s perceptions and experiences, shaped by their backgrounds, may explain disparities in accessing care and HRT prescriptions. Menopause can be highly disruptive for modern women as they combine high workloads and caring responsibilities distinct from previous generations. Participants referred to large amounts of products and services available and feeling overwhelmed when assessing their credibility, with some less-affluent participants feeling unable to afford them. Past discourses about HRT being dangerous remain deeply ingrained; however, understandings of risks vary, with some feeling confident in their understanding and others unsure.

Discussions with peers can act as a catalyst to initiate seeking health care, particularly HRT. During consultations, some felt frustrations related to not accessing HRT, being offered antidepressants, and a lack of approaches other than HRT being explored (for example, CAM or self-management). It was clear from women’s accounts that those who wanted HRT often believed they would have to advocate for this.

Cultural backgrounds shape approaches to managing menopause and influence women’s experiences of accessing care. As menopause is a natural process, South Asian and Black participants discussed how, within their communities, it is generally considered distinct from ailments warranting health care. They also noted how conversations about menopause were rare or non-existent. Some Black participants referred to historical mistrust of medical institutions and treatments. Lack of representation was problematic for participants from Black and Asian ethnicities, who said they were more likely to engage in material if someone like themselves was depicted, or if the conveyer of information was relatable. Lastly, some women were concerned about being stereotyped during consultations and modified their behaviour to temper this.

### Strengths and limitations

Use of qualitative focus groups and interviews allowed for in-depth exploration of women’s experiences of menopause and help-seeking. Public representatives were involved throughout all stages, including refinement of the study findings.

The sample was limited to those who were aware they were experiencing menopause and those who volunteered may have been experiencing severe symptoms. Although the sample included diverse perspectives, perspectives from other communities living in the UK are absent. Fieldwork was conducted in English, limiting consideration of the experiences of women who lacked fluency in English.

Generational comparisons were made based on difference perceived by participants and reinforced by the public representatives. However, direct experiences of older women were not present.

The findings are limited to women’s views, and we cannot comment on GPs’ rationales or experiences of consultations. Regardless of this, such perceptions are present and provide valuable insights into how care may be improved. The sister study provides further insights to intended delivery from clinicians’ perspectives.^16^

### Comparison with existing literature

Increasing awareness of menopause and HRT demand are well documented,^19,20^ as well as the subsequent pressure this has put on primary care.^19–21^ Reflecting this, our study shows how women often felt they had to advocate for HRT. However, demand for menopause care seems to be disproportionately from middle-class White women.^21^ Primary care staff recognise potential for low levels of awareness and self-advocacy related to menopause within ethnic minorities,^16,21^ and our findings add weight to this. However, less advocacy may not necessarily relate to low awareness, as our findings suggest women from Black and South Asian communities may be less inclined to seek HRT in favour of natural or alternative therapies. Women may feel they have to temper advocacy due to concerns about being stereotyped or upsetting their GP. The sister study examining clinicians’ perspectives showed how GPs often assume that women will self-advocate for HRT and that such discussions are typically not initiated with those who do not, noting the potential for missed opportunities.^16^ Our findings reinforce this, as we demonstrate how some women (who may benefit from HRT) do not initiate discussions for various reasons shaped by social and cultural influences. Mann *et al* ^16^ also showed how clinicians had noted recent changes in public awareness and highlighted issues around communication about treatment options. This is also congruent with our findings, as the heightened awareness was evident in women’s accounts, and many felt frustrated with the treatment options (particularly non-hormonal options) presented to them, suggesting that discussions about treatments could be improved.

### Implications for research and practice

While there is greater awareness and self-advocacy for menopause care, previous research^16,21^ and HRT prescribing rates^12,13^ indicate this is not experienced equally and our findings demonstrate reasons why. Rather than assuming women will self-advocate, sensitively initiating discussions about menopause and options available, particularly with women from lower socioeconomic and ethnic minority backgrounds, might improve access to care. Participants expected, or found, GPs’ advice outside of being offered HRT to be lacking. Lifestyle advice and social prescribing could help some women manage menopause symptoms. Concerns about lack of representation are well founded as women from ethnic minorities are underrepresented within menopause research.^22^ This study emphasises the importance of representation within information and future research to ensure content is relatable to all women.

In a context of heightened social media and commercialisation, women discussed feeling overwhelmed and uncertain about content credibility. GPs can play an important role in providing clear and accurate information to women to equip them in making sense of information and support informed decisions.^23^ This issue becomes more salient when supporting women from South Asian and Black communities, as our study suggests they may be more inclined to look to alternatives to manage their menopause. However, when women’s needs are not met within the primary care consultation, they are likely to look to alternative products, approaches, and private clinics for solutions.^19^

For women that do consult about menopause, our findings demonstrate key issues. It is problematic that many feel they must advocate for access to HRT and that some are unsure about risks. Menopause consultations would benefit from clear and robust discussions about all the options available and their associated risks for that individual, with the option of HRT.
